# Cytomegalovirus enterocolitis with subsequent diagnosis of coexisting new-onset inflammatory bowel disease: Two case reports and review of the literature

**DOI:** 10.1097/MD.0000000000024914

**Published:** 2021-02-26

**Authors:** Panupong Luangsirithanya, Sukrit Treewaree, Ananya Pongpaibul, Nonthalee Pausawasdi, Julajak Limsrivilai

**Affiliations:** a Division of Gastroenterology, Department of Medicine; b Department of Pathology, Faculty of Medicine Siriraj Hospital, Mahidol University, Bangkok, Thailand.

**Keywords:** case report, cytomegalovirus enterocolitis, gastrointestinal-cytomegalovirus, inflammatory bowel disease, new-onset inflammatory bowel disease

## Abstract

**Introduction:**

Gastrointestinal (GI) cytomegalovirus (CMV) infection coexisting with or followed by a diagnosis of inflammatory bowel disease (IBD) is infrequently reported. Not recognizing this condition may delay IBD diagnosis in patients with GI-CMV disease who do not or partially respond to antiviral agents, which could consequently result in unsatisfied treatment outcomes.

**Patient concerns:**

Two immunocompetent patients with no known underlying GI conditions presented with acute bloody diarrhea. The first patient developed diarrhea and hematochezia after admission to intensive care unit (ICU) because of severe alcoholic pancreatitis for 10 days duration. Computed tomography abdomen showed segmental jejunal thickening. The other patient presented with a 1-week history of severe bloody diarrhea which required ICU admission. Colonoscopy showed multiple ulcers along terminal ileum and colon.

**Diagnosis:**

These 2 patients were initially diagnosed with CMV jejunitis and ileocolitis, respectively, based on endoscopic and histopathologic findings. Both had partial response to treatment with 3 weeks of intravenous ganciclovir. Crohn disease was suspected because of persistent ulcerations on the follow-up endoscopy with the presence of pathological features of chronic inflammation and disappearance of previously detected CMV-infected cells.

**Intervention:**

Both patients were treated with systemic corticosteroids and azathioprine.

**Outcomes:**

Both patients had complete clinical improvement. Prednisolone could be tapered off in 6 months. Follow-up video capsule endoscopy (VCE) at 6 months showed improvement of mucosal inflammation and ulcers, but neither were completely healed in the first patient. Follow-up colonoscopy at 6 months showed complete resolution of ulcers and inflammation in the second patient.

**Lessons:**

IBD should be suspected in patients with a diagnosis of GI-CMV disease who are immunocompetent and have a partial response to antiviral agents. This clinical scenario could be caused by either CMV infection activating immune response resulting in IBD onset, or CMV infection superimposed on pre-existing latent IBD.

## Abstract 2

Supplemental Digital Content is available in the text

## 1 Introduction

Cytomegalovirus (CMV) is an enveloped double-stranded DNA virus in Herpesviridae family.^[1–3]^ Primary infection is common in human population with a 40% to 100% of seroprevalence rate, and those affected may be either symptomatic or asymptomatic.^[4–6]^ Like other viruses in the Herpesviridae family, CMV infection can be lifelong. Lifelong latent infection can be found in various cell types, including hematopoietic cells, endothelial cells, and fibroblasts.^[7,8]^ Reactivation is most likely to occur when host immune function is decreased or suppressed.

The association between CMV and inflammatory bowel disease (IBD) was first recognized and reported in 1961.^[9]^ It was also reported that CMV can potentiate disease activity resulting in more aggressive disease and worse outcomes.^[10]^ Standard practice guidelines recommend investigation for gastrointestinal (GI) CMV infection in patients with IBD who are refractory to treatment.^[11]^ Interestingly, there have been some case reports of GI-CMV infection presenting as the first manifestation of IBD. This clinical scenario could be caused by either CMV infection activating immune response resulting in IBD onset, or CMV infection superimposed on pre-existing latent IBD. Patients usually present with partially improved or unimproved enterocolitis after treatment for GI-CMV infection. Although this condition is uncommon, under-recognition could lead to misdiagnosis of IBD with refractory GI-CMV disease, resulting in unnecessary treatment with antiviral therapy and delayed IBD diagnosis. Notably, no study has collectively analyzed and described the clinical characteristics, investigations, treatments, and treatment outcomes of patients with these 2 coexisting conditions. Accordingly, we performed a systematic review of previously reported cases and analyzed them together with our two cases of GI-CMV disease—both of which occurred prior to diagnosis of IBD.

## 2 Methods

### 2.1 Case 1

A 51-year-old Thai man with a 30-year history of chronic alcohol consumption was referred to our hospital due to mid gastrointestinal bleeding. Three weeks before referral, he was admitted to a local hospital due to hypotension and severe vomiting >10 episodes per day. The initial laboratory workup found elevated serum amylase (318 U/L), elevated serum creatinine (5.3 mg/dL), and decreased platelet count (82 000/μL). He was diagnosed with acute severe alcoholic pancreatitis, which required intensive care unit (ICU) admission with ventilatory support, inotropes, and hemodialysis. Ten days later, his hemodynamic and respiration problems were improved, but he started having watery diarrhea, fever, and abdominal pain. During the next day, his stool changed color to maroon-colored stool. His hematocrit decreased from 29.5% to 23%. He underwent esophagogastroduodenoscopy (EGD) and colonoscopy, but the findings were normal, except for blood in the colon and terminal ileum. Computed tomography (CT) angiography revealed long-segment jejunal thickening and dilatation with active contrast extravasation. He was then referred to our center.

**Figure 1:**
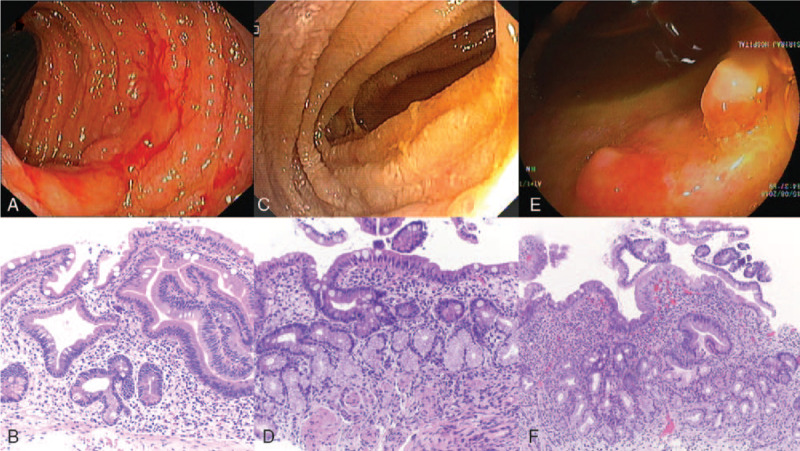
Endoscopic and pathologic findings of case 1. The initial endoscopic and pathologic findings are shown in A and B, respectively. Endoscopic findings showed multiple large clean-base ulcers surrounded by markedly inflamed mucosa. Histopathology showed ulceration, focal blunt villi, and cytomegaloviral inclusion bodies. Features of chronic enteritis, including dense chronic inflammatory infiltration and pyloric metaplasia, were not observed. C and D are the findings of endoscopy after 3 weeks of ganciclovir treatment. Those findings showed healing of ulcers with improvement of mucosal inflammation; however, the histopathologic findings revealed dense chronic inflammatory infiltration in submucosa with presence of pyloric metaplasia. Viral inclusion was not seen. E and F show the findings 6 months later whtn the patient had clinical relapse. Endoscopic findings showed recurrence of ulcers with granulation nodules at surrounding mucosa, and the pathologic findings showed more obvious features of chronic enteritis without viral inclusion.

During admission at our hospital, he still had epigastric pain and passed about 500 to 600 mL of melena per day. Abdominal examination showed mild tenderness at epigastrium. His initial laboratory tests were, as follows: hemoglobin (Hb) 10.8 g/dL, blood urea nitrogen (BUN) 63.5 mg/dL, creatinine (Cr) 2.29 mg/dL, albumin (Alb) 2.2 g/dL, globulin (Glb) 5.2 g/dL, serum lipase 257 U/L, and prothrombin time (PT) 14.8 seconds. Antegrade enteroscopy was performed 14 days after onset of diarrhea, which found multiple large clean-base ulcers with contact bleeding surrounded by inflamed mucosa (Fig. 1A). The pathology showed ulceration, focal blunt villi, and cytomegaloviral inclusion bodies. Features of chronic enteritis, such as dense chronic inflammatory infiltration and pyloric metaplasia, were not observed (Fig. 1B). He was treated with intravenous ganciclovir 600 mg per day for 3 weeks with clinical improvement. His diarrhea and GI bleeding stopped. Follow-up enteroscopy showed improvement of enteritis (Fig. 1C), and no CMV inclusion was observed in pathologic findings. However, some chronic features of enteritis were identified (Fig. 1D). Despite these chronic enteritis features, he was able to be discharged to his home. At 3 months after discharge, he had neither diarrhea nor GI bleeding, and his hemoglobin level was increased to 13 g/dL. However, he still had intermittent abdominal pain, and he had not regained the 10 kg that he lost during the last hospitalization. Furthermore, his albumin level increased to only 2.7 g/dL.

Six months after discharge, his symptoms relapsed. He passed watery and mucous bloody stools 4 to 5 episodes a day, and he lost an additional 5 kg of body weight. His hemoglobin dropped to 8 g/dL, and his albumin level decreased to 1.7 g/dL. He was readmitted and underwent antegrade enteroscopy, which revealed more ulcers compared with the findings of the last endoscopy (Fig. 1E). The pathologic study found progression of chronic enteritis with marked activity, but no viral inclusion, granuloma, or malignancy (Fig. 1F). Ganciclovir was given despite negative viral inclusion. On day 7 of ganciclovir, his symptoms were progressively worsening. Since chronic enteritis was noted in tissue specimens, jejunal Crohn disease was suspected. He was then treated with prednisolone 40 mg per day, and his clinical was dramatically improved. Abdominal pain and diarrhea resolved within 2 weeks and he was discharged from the hospital. On follow-up, azathioprine 50 mg per day was started and titrated up to 100 mg per day, and prednisolone was tapered and discontinued within 6 months. By the 6-month follow-up, all symptoms had resolved and he had regained the 15 kg of body weight that he had lost. His hemoglobin and albumin levels returned to normal at 14 and 4.2 g/dL, respectively. Follow-up video capsule endoscopy (VCE) showed improvement of mucosal inflammation and ulcers, but neither were completely healed.

### 2.2 Case 2

**Figure 2:**
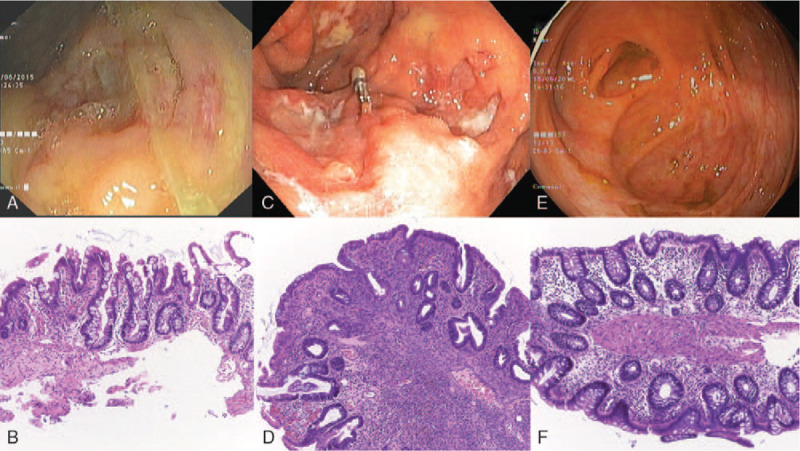
Endoscopic and pathologic findings of case 2. A and B represent the findings of the first endoscopy. Endoscopic findings showed large ulcers on inflamed mucosa. The histology showed chronic active inflammation with presence of viral inclusion. Some chronic enterocolitis features, including dense chronic inflammatory infiltration in mucosa and submucosa with crypt branching and crypt dropout, were observed. The findings of follow-up colonoscopy after 3 weeks of ganciclovir treatment are shown in C and D. Those findings revealed worsening of endoscopic features and more dense chronic inflammatory infiltration. E and F demonstrate the findings after remission was achieved after treatment with corticosteroid and azathioprine. The colonic mucosa looked normal in both endoscopic and histologic findings.

A 49-year-old Thai woman with underlying diseases of hypertension and migraine headache was admitted to the ICU due to severe enterocolitis with septic shock, hypoglycemia, acute renal failure with severe metabolic acidosis requiring hemodialysis, and respiratory failure requiring mechanical ventilator. She began to have mucous bloody diarrhea 5 to 6 episodes per day with vomiting and generalized abdominal pain with fever 1 week prior to admission. Abdominal examination showed generalized mild tenderness with hypoactive bowel sounds. Her initial laboratory findings were, as follows: Hb 15.7 g/dL, white blood count (WBC) 29 000/μL, platelet count 465,000/μL, sodium (Na) 112 mEq/L, potassium (K) 7.9 mEq/L, bicarbonate (HCO_3_^−^) 2 mEq/L, BUN 132.4 mg/dL, Cr 5.31 mg/dL, albumin 3.7 g/dL, globulin 5.3 g/dL, lactate 3.9 mmol/L, and creatine kinase (CPK) 323 U/L. Stool examination found WBC 50 to 100/HPF with no growth of bacterial pathogens on stool culture. She was treated with intravenous ceftriaxone and metronidazole. Five days later, she developed worsening abdominal pain, passed maroon-colored stool, and had retained bilious gastric content. CT whole abdomen showed diffuse small and large bowel dilatation suggestive of bowel ileus. Antibiotic was changed to intravenous meropenem. After 3 weeks of treatment, her hemodynamic and respiration parameters were improved, but diarrhea was still ongoing with 1000 to 2000 mL of stool per day accompanied by intermittent hematochezia. She required packed red cells 1 to 2 units per week, and her serum albumin level dropped to 1.7 mg/dL. EGD and colonoscopy were performed, and diffuse ulcerated edematous mucosa from the sigmoid colon to the terminal ileum was found (Fig. 2A). The pathology demonstrated CMV immunostaining positive cells from her left-side and right-side colon with some chronic colitis features (Fig. 2B). Ganciclovir 400 mg/day was administered intravenously for 3 weeks. Her mucous bloody diarrhea was improved, but she still had watery diarrhea and fever. Follow-up colonoscopy after 3 weeks of ganciclovir treatment found larger and deeper ulcers, which were prominent at the cecum and sigmoid colon (Fig. 2C). The pathologic study found neither viral inclusion nor CMV immunohistochemistry (IHC) positive cells, but marked chronic active inflammation was observed (Fig. 2D).

She was then treated as Crohn disease with intravenous dexamethasone 15 mg per day. Her clinical improved dramatically, her fever disappeared, and her stool became solid in 2 weeks. Her serum albumin level increased to 2.6 g/dL, and her treatment was changed to prednisolone 40 mg/day and azathioprine 50 mg/day. She was then discharged from the hospital. During follow-up, she remained in clinical remission, and her albumin level returned to normal at 4 mg/dL in 3 months. Prednisolone was tapered and discontinued in 6 months, and only azathioprine 50 mg/day was continued to maintain remission. Follow-up colonoscopy was performed, which showed that all ulcers had completely healed (Fig. 2E), and her histopathologic findings had also returned to normal (Fig. 2F).

### 2.3 Systematic review of previously reported cases

A systematic search was performed from inception to January 2020 using the databases from PubMed and Embase to identify previous case reports or case series of new-onset IBD following or IBD with coexisting GI-CMV infection. The search terms that we used were: “Cytomegalovirus” AND (“Inflammatory bowel disease” OR “Crohn” OR “Ulcerative colitis”). Only articles that provided clinical information, including clinical presentation, laboratory investigations, endoscopy, and histology for each patient, were included. GI-CMV disease needed to be confirmed by presence of inclusion body or positive immunohistochemistry IHC in tissue specimens. IBD diagnosis was based on clinical, endoscopic, and histologic findings, and needed to be confirmed by response to treatment. Immunocompromised patients or patients taking immunosuppressive agents were excluded from the study. Eligible articles were reviewed by 2 authors independently (PL and ST). Any disagreement was resolved by discussion and consensus, or with the input of a third author (JL), as needed. Studies meeting our inclusion criteria were included, and the data from those studies were analyzed together with the data from our 2 cases. The protocol for this study was approved by the institutional review board of our hospital. Written inform consent was obtained from both of our patients.

### 2.4 Statistical analysis

Continuous variables are expressed as mean ± standard deviation or median and interquartile range, as appropriate. Categorical variables are expressed as the number of subjects and percentages. SAS Statistics software (SAS, Inc., Cary, NC) was used for all statistical analyzes.

## 3 Results

**Figure 3:**
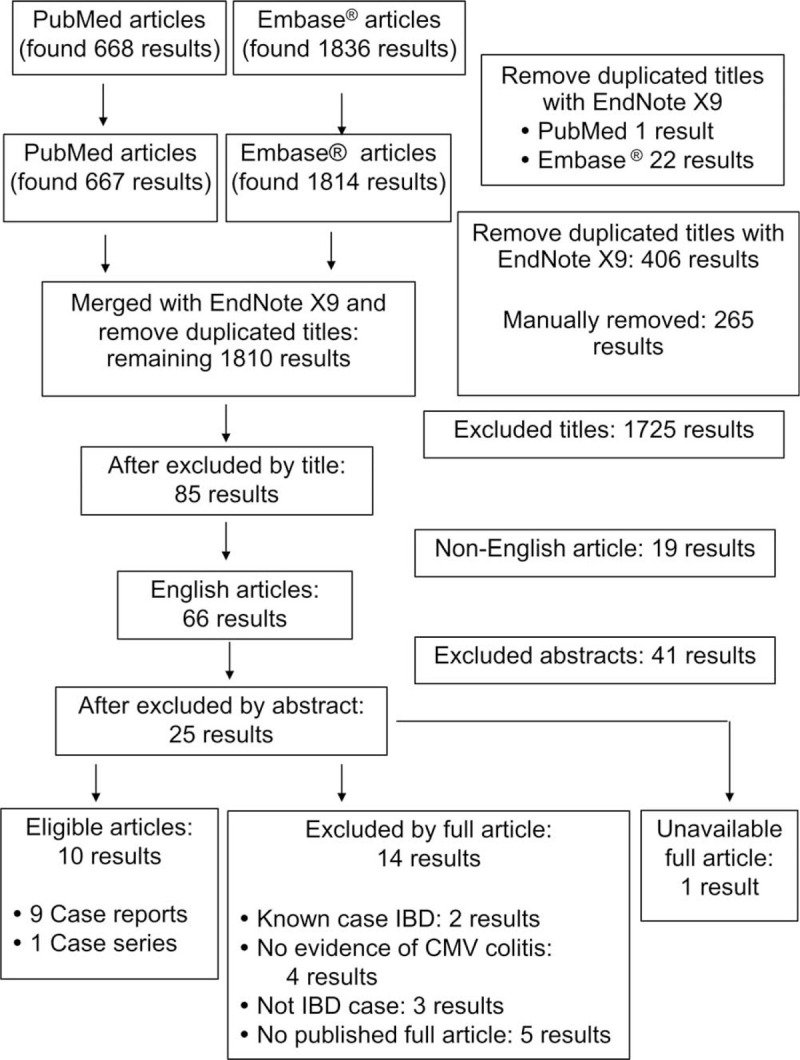
The systematic literature review process.

The systematic search yielded 11 cases from 9 case reports,^[9,12–19]^ and 1 case series^[20]^ from 1961 to 2017. Fourteen articles were excluded for the following reasons. Two articles^[21,22]^ included known cases of IBD; 4 articles^[23–26]^ includes patients with CMV infection diagnosed by positive serology, but CMV was not detected in colonic mucosa; 3 articles^[27–29]^ included patients presenting with CMV colitis, but IBD was not confirmed by response to IBD treatment; and, 5 articles^[30–34]^ had only an abstract that lacked a sufficient amount of clinical information. One article^[35]^ was further excluded due to our inability to access the full article. The systematic literature review process is shown in Fig. 3. In Table 1, we summarized the findings of our cases and previously reported cases. The findings detail was shown in supplementary Table 1.

**Table 1:** Summary of findings from patients with new-onset inflammatory bowel disease (IBD) and coexisting cytomegalovirus (CMV) colitis from previously reported cases and our cases.

	Previous cases		Our cases		Total cases	
Characteristics	n	Result	n	Result	n	Result
Age, mean ± SD (range)	11	40.3 ± 13.4 (18–61)	2	49 and 51	13	41.8 ± 12.8 (18–61)
Gender	11		2		13	
Male		4 (36%)		1 (50%)		5 (38%)
Female		7 (64%)		1 (50%)		8 (62%)
Duration of presentation (days), median (IQR)	10	12 (7–28)	2	7 and 21 Median 14	12	12 (7–23)
Clinical	11		2		13	
Fever		10 (91%)		2 (100%)		12 (92%)
Body temp, °C, mean ± SD, (range)	9^∗^	39.0 ± 0.6 (38.3–40.0)		37.8 and 39.9 Mean 38.8	11^∗^	39.0 ± 0.7 (37.8–40.0)
Diarrhea						
Only watery		4 (36%)		0 (0%)		4 (31%)
Bloody (±watery)		7 (64%)		2 (100%)		9 (69%)
Nausea and/or vomiting		0 (0%)		2 (100%)		2 (15%)
Abdominal pain and/or tenderness		8 (73%)		2 (100%)		10 (77%)
Investigation						
Hemoglobin level (g/dL), mean ± SD (range)	7^†^	10.6 ± 1.1 (8.8–12.0)	2	10.8, 15.7 (at presentation) 7.7, 6.0 (lowest values)	9^†^	11.2 ± 1.9 (8.8–15.7)
Albumin level (g/dL) mean ± SD (range)	2	2.9 [9], 3.3 [16] (at presentation)	2	2.2, 3.7 (at presentation) 1.3, 1.2 (lowest values)	4	3.0 ± 0.6 (2.2–3.7)
CMV IgM positive	9	7 (78%)	0	Not tested	9	7 (78%)
CMV IgG positive	8	5 (62%)	0	Not tested	8	5 (62%)
Blood CMV viral load	1	3200 copies/mL	2	<20 copies/mL	3	Cannot be calculated
Gross findings	11		2		13	
Inflamed mucosa		10 (91%)		2 (100%)		12 (92%)
Ulcer		5 (45%)		2 (100%)		7 (54%)
Pathologic findings						
IHC positive for CMV	7	7 (100%)	2	2 (100%)	9	9 (100%)
CMV inclusion body	11	8 (72.7%)	2	2 (100%)	13	10 (76.9%)
Treatment	11		2		13	
Ganciclovir		8 (73%)		2 (100%)		10 (77%)
Corticosteroid		6 (55%)		2 (100%)		8 (62%)
Azathioprine		0 (0%)		2 (100%)		2 (15%)
5-ASA (mesalamine)/sulfasalazine		6 (55%)		1 (50%)		7 (54%)
Result of treatment	11		2		13	
Cases that started with CMV treatment	5		2		7	
No improvement after CMV treatment alone		2 (40%) [18, 20]		0 (0%)		2 (29%)
Partial improvement after CMV treatment alone		1 (20%) [20]		2 (67%)		3 (42%)
Complete clinical improvement after CMV treatment alone		2 (40%) [13, 16]		0 (0%)		2 (29%)
Complete clinical improvement after IBD treatment after free from CMV colitis		N/A		2 (100%)		2 (100%)
Cases that started with IBD treatment	4		0		4	
No improvement and finally required surgery		1 (25%) [9]				1 (25%)
Partial improvement after IBD treatment alone		1 (25%) [15]				1 (25%)
Complete clinical improvement after IBD treatment alone		2 (50%) [14, 19]				2 (50%)
Cases that started with both CMV and IBD treatment	2		0		2	
Complete clinical improvement after started with both CMV and IBD treatment		2 (100%) [12, 17]				2 (100%)
Type of IBD	11		2		13	
Crohn disease		1 (9%)		2 (100%)		3 (23%)
Ulcerative colitis		8 (73%)		0 (0%)		8 (62%)
Unidentified		2 (18%)		0 (0%)		2 (15%)

n = number of cases with available data. A week was converted to 7 days and a month was converted to 30 days. °C = degrees Celsius, CMV = cytomegalovirus, IBD = inflammatory bowel disease, IgG = immunoglobin G, IgM = immunoglobin M, IHC = immunohistochemistry, IQR = interquartile range, N/A = not available, SD = standard deviation.

∗ One case was excluded due to body temperature reported as greater than 38 °C.

† Two cases were excluded due to hemoglobin level reported as hematocrit level.

New-onset IBD associated with CMV colitis was reported in patients aged 18 to 61 years in both sexes. The majority (62%) of IBD were ulcerative colitis. The common presenting symptoms included high-grade fever (92%), abdominal pain (77%), and diarrhea (100%). The duration of symptoms prior to diagnosis was 7 to 23 days. The average Hb level at presentation was 11.2 g/dL. Serum CMV IgM and IgG could be found in most previously reported cases; however, these parameters were not tested in our cases. The gross pathologic findings included inflamed mucosa and multiple ulcers. Ten of 13 cases reported CMV inclusion bodies found in tissue. Seven previously reported cases were IHC positive for CMV, while the other 4 cases were not tested by this technique.

Regarding treatment (shown in Table 1), 7 of 13 (54%) patients were initially treated with intravenous ganciclovir. Of those, 2 patients had complete clinical improvement, but still had active IBD on follow-up colonoscopy. The remaining 5 patients with either no or partial response to intravenous ganciclovir required IBD treatment to achieve clinical remission. Four of 13 (30.7%) patients were initially treated with IBD medications. Of those, 2 achieved complete clinical remission, 1 achieved partial clinical remission, and 1 had no improvement and finally required surgery. The 2 patients with complete remission by IBD treatment alone were diagnosed GI-CMV disease based on positive IHC in colonic tissue, but negative for viral inclusion. The other 2 of 13 (15.3%) patients received intravenous ganciclovir and IBD medications at the same time, and both had complete clinical improvement.

## 4 Discussion

The association between CMV colitis and IBD has been recognized since Powell et al^[9]^ reported a patient with ulcerative colitis who was found to have CMV inclusion in colonic tissue. Although CMV enterocolitis in IBD usually occurs among those who received corticosteroids or immunosuppressive agents,^[36,37]^ our 2 cases and the other included previously reported cases uncommonly had not been diagnosed with IBD when they developed GI-CMV disease. This raises the question of whether CMV caused or triggered IBD, or whether IBD was the true cause of enterocolitis with CMV superinfecting an already inflamed mucosa. This issue is still inconclusive. Our first case supports the hypothesis that CMV infection might cause IBD since the first pathologic findings did not show any chronic enteritis features, but these chronic features were clearly demonstrated on the later pathologic specimens. Some evidence suggests that CMV infection may increase the risk of IBD development. Onyeagocha et al^[38]^ found that CMV infection in mice altered intestinal mucosal immunity, which resulted in an increased tendency of CMV-infected hosts to develop dextran sodium sulphate-induced colitis. Furthermore, viral proteins of CMV may upregulate the immune system and induce major histocompatibility complex class I surface antigen expression that could promote inflammation.^[39]^ Moreover, a clinical study by Verdonk et al^[40]^ showed that de novo IBD occurred more often in orthotopic liver transplanted patients who experienced CMV infection in the postoperative period when compared with those did not have CMV infection. On the other hand, our second case supports the hypothesis that CMV infection might occur on top of latent IBD since the first pathologic findings demonstrated chronic colitis features. Other evidence supports that IBD milieu increased the risk of CMV reactivation. Hommes et al^[41]^ proposed that proinflammatory cytokines from IBD, including tumor necrosis factor-α, interferon-γ, and interleukin-2, can induce expression of chemokines and transcription molecules resulting in recruitment of monocytes and dendritic cells, the cells that could be latently infected by CMV. This mechanism promotes CMV reactivation, which stimulated T-cell and further inflammation.

The common clinical manifestations of patients with CMV enterocolitis with coexisting IBD in our patients and previous case reports were subacute presentation of fever, diarrhea, hematochezia, and abdominal pain. Typically, GI-CMV alone is common among immunocompromised patients; however, could be found in immunocompetent patients as well. Nonetheless, patients commonly have some underlying comorbid illnesses such as critically-ill setting or old age.^[42]^ Patients with IBD alone could also present with the same symptoms; however, high-grade fever, which was observed in most patients with CMV infection, was not frequently observed in IBD without complications.^[43,44]^

The endoscopic findings of GI-CMV disease have a wide spectrum. They could range from mild and patchy erythematous and edematous mucosa with subepithelial hemorrhage to large ulcerations and pseudomembrane.^[45]^ These findings can mimic either ulcerative colitis or Crohn disease. In ulcerative colitis, if specific endoscopic findings and location, including diffuse inflammation starting from rectum and extending proximally, are observed, but punch-out lesions, which are found in CMV colitis, are also present, ulcerative colitis with superimposed CMV infection should be suspected.^[46]^ In Crohn disease, the endoscopic findings are difficult to differentiate from GI-CMV disease. Diagnosis, therefore, requires presence of organisms in intestinal tissue. The visualization of cytomegalic cells in a mucosal biopsy by Hematoxylin and Eosin (H&E) staining can be used for diagnosis of GI-CMV disease with high specificity, but low sensitivity. The sensitivity in this study was only 77%. Immunohistochemistry and tissue polymerase chain reaction (PCR) help to increase the sensitivity. The pathologic findings may be helpful for identifying patients with GI-CMV disease who are likely to have concomitant IBD. Chronic injury findings, such as crypt architectural change, should heighten physician awareness for coexisting IBD in CMV-infected patients, as described in our case 2.

In IBD patients with concomitant GI-CMV infection, therapy for CMV eradication is recommended because the patients almost eventually need to take immunosuppressive agents for control their IBD.^[37]^ However, response to antiviral agents will depend on whether CMV infection is the main driving mechanism for inflammation in these patients. In 5 previous case reports and in both of our patients, ganciclovir was given initially, and all of them had at least partial response to treatment. Two patients had complete clinical improvement despite ongoing colitis on endoscopy. One patient who initially received IBD therapy had clinical deterioration that ultimately required colectomy.^[9]^ CMV infection was likely the main driving mechanism for inflammation in these 8 patients. On the other hand, 3 previously reported patients initially received treatment for IBD, and they also had at least partial response to therapy.^[14,15,19]^ Two of those patients had complete resolution of symptoms without requiring antiviral therapy.^[14,19]^ We observed that the diagnosis of GI-CMV disease in those 2 patients was based on IHC, while no viral inclusion was detected by H&E stain in the pathologic specimens. This may reflect a low viral load in the tissue in these 2 patients. High CMV viral load in intestinal tissue was reported to be a significant predictor of response to antiviral agents in IBD patients.^[47]^

## 5 Conclusion

IBD may be diagnosed following a diagnosis of GI-CMV disease. IBD should be suspected in patients with a diagnosis of GI-CMV disease who are immunocompetent and who have no or partial response to antiviral agents. This clinical scenario could be caused by either CMV infection activating immune response resulting in IBD onset, or CMV infection superimposed on pre-existing latent IBD.

## Acknowledgment

The authors gratefully acknowledge the patients described in this report for permitting us to disclose details and images relating to their cases.

They also gratefully acknowledge Asst. Prof. Kevin P. Jones, Medical Research Manuscript Editor, Siriraj Medical Research Center (SiMR), Faculty of Medicine Siriraj Hospital, Mahidol University for language editing.

## Author contributions

**Conceptualization:** Julajak Limsrivilai.

**Data curation:** Panupong Luangsirithanya.

**Formal analysis:** Panupong Luangsirithanya, Sukrit Treewaree, Julajak Limsrivilai.

**Investigation:** Panupong Luangsirithanya, Julajak Limsrivilai.

**Project administration:** Julajak Limsrivilai.

**Resources:** Ananya Pongpaibul, Julajak Limsrivilai.

**Software:** Julajak Limsrivilai.

**Supervision:** Julajak Limsrivilai.

**Writing – original draft:** Panupong Luangsirithanya, Sukrit Treewaree, Julajak Limsrivilai.

**Writing – review & editing:** Ananya Pongpaibul, Nonthalee Pausawasdi, Julajak Limsrivilai.

## Supplementary Material

Supplemental Digital Content
